# FYVE-Dependent Endosomal Targeting of an Arrestin-Related Protein in Amoeba

**DOI:** 10.1371/journal.pone.0015249

**Published:** 2010-12-13

**Authors:** Dorian Guetta, Karine Langou, Didier Grunwald, Gérard Klein, Laurence Aubry

**Affiliations:** 1 CEA, DSV, iRTSV, Laboratoire de Biochimie et Biophysique des Systèmes Intégrés, Grenoble, France; 2 LBBSI, UMR5092 CNRS, Grenoble, France; 3 Université Joseph Fourier, Grenoble, France; 4 CEA, DSV, iRTSV, Lab. Transduction du Signal, Grenoble, France; 5 LTS, EMI104 INSERM, Grenoble, France; Iowa State University, United States of America

## Abstract

**Background:**

Visual and β-arrestins are scaffolding proteins involved in the regulation of receptor-dependent intracellular signaling and their trafficking. The arrestin superfamilly includes several arrestin domain-containing proteins and the structurally related protein Vps26. In *Dictyostelium discoideum*, the arrestin-domain containing proteins form a family of six members, namely AdcA to -F. In contrast to canonical arrestins, *Dictyostelium* Adc proteins show a more complex architecture, as they possess, in addition to the arrestin core, other domains, such as C2, FYVE, LIM, MIT and SAM, which potentially mediate selective interactions with either lipids or proteins.

**Methodology and Principal Findings:**

A detailed analysis of AdcA has been performed. AdcA extends on both sides of the arrestin core, in particular by a FYVE domain which mediates selective interactions with PI(3)P, as disclosed by intrinsic fluorescence measurements and lipid overlay assays. Localization studies showed an enrichment of tagged- and endogenous AdcA on the rim of early macropinosomes and phagosomes. This vesicular distribution relies on a functional FYVE domain. Our data also show that the arrestin core binds the ADP-ribosylation factor ArfA, the unique amoebal Arf member, in its GDP-bound conformation.

**Significance:**

This work describes one of the 6 arrestin domain-containing proteins of *Dictyostelium*, a novel and atypical member of the arrestin clan. It provides the basis for a better understanding of arrestin-related protein involvement in trafficking processes and for further studies on the expanding roles of arrestins in eukaryotes.

## Introduction

The plasma membrane represents the interface between the cell interior and the extracellular environment. Appropriate physiological responses to external stimuli rely on receptors, transporters and other intrinsic protein equipment located at the membrane boundary. The activity of some of these protein families, of which the GPCRs have been described in most detail, is modulated by integrated activation mechanisms followed by downstream signaling, desensitization and resensitization/recycling or degradation. Endocytosis is a major mechanism involved in the attenuation-resensitization process of several ligand-activated GPCRs. By binding to phosphorylated GPCRs, the β-arrestins not only block heterotrimeric G-protein dependent signaling by preventing access to G-proteins but also initiate the first steps of receptor internalization in clathrin-coated vesicles through the recruitment of clathrin and adaptor proteins. While promoting the desensitization and endocytosis of membrane targets, β-arrestins have also been shown to activate downstream signaling cascades by locally controlling the activity of kinases (Src and MAPK) and other enzymes. Additionally, it is now well established that the roles of β-arrestins extend to membrane proteins other than GPCRs, broadening the field of action for those scaffolding proteins known to play a critical role in vesicular membrane trafficking and cell signaling [1]–[4]. Although most arrestin literature emanated from the study of mammalian proteins, important in vivo and mechanistic insights have been unveiled in alternative models such as flies, zebrafish, worms and more recently fungi [5]–[12].

The social soil amoeba *Dictyostelium discoideum* is an attractive model system for use in studying the regulation of membrane trafficking events: it is a genetically tractable organism with highly active endocytic functions. Endocytosis ensures efficient entry of nutrients either by macropinocytosis or by phagocytosis [13]. Due to their endocytic activity, cells internalize the equivalent of their entire cell surface every 45 min. Although it has been well illustrated that integral proteins from the plasma membrane have different fates both during endocytic vesicle formation (exclusion *vs* internalization) and once they have been internalized (recycling or not), little is known about the sorting events in these early steps of endocytosis [14]. The *Dictyostelium* genome encodes six arrestin-related proteins (AdcA to F) as candidates for a putative function in the sorting of membrane proteins [15]. This report presents the characterization of one of them, the novel protein AdcA in which the arrestin core found alone in canonical arrestins is here part of a more complex architecture and functions in association with supplementary modules including a FYVE domain that mediates selective interactions with endosomal PI(3)P.

## Materials and Methods

### Materials

Mouse monoclonal antibodies against p80, p25 and vacuolin were kind gifts from Pierre Cosson (University of Geneva, Switzerland) and Markus Maniak (University of Kassel, Germany) [16], [17]. Anti-actin and anti-cathepsin D antibodies were generously provided by Jérôme Garin (CEA-Grenoble, France) [18]. Anti-GFP and anti-myc 9E10 monoclonal antibodies were purchased from Roche Biochemicals (Meylan, France), and the rabbit anti-MBP antiserum from New England Biolabs (Ozyme, Saint-Quentin-en-Yvelines, France). The rabbit anti-Arf monoclonal antibody (clone ID EP442Y) directed against a peptide of Arf1 conserved in Dd-ArfA was purchased from Epitomics (Fermentas, France). HRP-conjugated secondary antibodies were purchased from Bio-Rad (Marnes-la-Coquette, France) and Alexa Fluor 488- and Cy3-conjugated secondary antibodies from Molecular Probes and Jackson ImmunoResearch respectively (Interchim, Montluçon, France). Texas Red-conjugated zymosan A *Saccharomyces cerevisiae* BioParticles®, tetramethylrhodamine-conjugated *Escherichia coli* BioParticles® and DAPI were obtained from Molecular Probes. PIP strips and Ins(1,3)P_2_ were purchased from Echelon Biosciences Inc. (Tebu-bio, Le-Perray-en-Yvelines, France). LY294002 (50 mM stock in DMSO) was obtained from Sigma (Saint-Quentin Fallavier, France).

### Plasmid constructs

For overexpression purposes, most of AdcA-derived constructs were subcloned in the *Bgl*II-*Xho*I sites of Exp4+ (neoR) under the control of the *actin15* promoter and tagged with green fluorescent protein GFP or with a double-myc epitope at the C-terminus except when mentioned otherwise. The following constructs were generated: AdcA (amino acid 1 to 580), AdcAΔHФ (amino acid 140 to 580), AdcAΔFY (amino acid 1 to 459), AdcAΔF (amino acid 1 to 460 and 531 to 580), FY (amino acid 458 to 580), FYVE (amino acid 458 to 530), AdcA^R491A^ (amino acid 1 to 580). This point-mutant was generated by PCR using oligonucleotides carrying the Arg to Ala mutation in position 491 as well as a silent mutation introducing a *Bgl*II site to facilitate subcloning. AdcA was also expressed as an mRFPmars fusion protein with the tag introduced on the N-terminal side. For this, full-length AdcA was subcloned in the mRFPmars vector that includes a blasticidin resistance cassette (bsR) [19]. The *adcA* knockout strain was generated by targeted integration of the *bsr* cassette at position 496 of the *adcA* gene. For biochemical analysis purposes, the FYVE domain (amino acid 458 to 530) and the H domain (amino acid 1 to 116) were subcloned in pMAL-C2 in fusion with MBP (sites *Bam*HI/*Sal*I) and pET-duet1 (sites *Nde*I/*Xho*I, no tag) respectively. *Dictyostelium arfA* (DDB_G0289173) was subcloned in pET28 in frame with the C-terminal His_6_ tag and AdcA_C_ (C domain of the arrestin core, amino acid 311 to 459) in pGEX-KG in frame with the N-terminal GST. All the constructs that required PCR amplification were verified by sequencing (Cogenics, Grenoble, France).

### Cell culture, knockout and development

*D. discoideum* parental strain KAx-3 and derived mutants were grown at 22°C in axenic medium in shaking suspension or in plastic Petri dishes [20]. *Dictyostelium* cells were transfected by electroporation. Overexpressors were selected by addition of G418 (20 µg/ml) or blasticidin (7.5 µg/ml) depending on the expression vector. The *adcA* null cells were selected in the presence of blasticidin and cloned by plating transformants onto SM-agar plates in association with *Klebsiella aerogenes*. Disruption of *adcA* was verified by Southern blot and Western blot analyses. Development was induced by plating cells on non-nutritive Na,K-Pi-buffered agar plates [21], [22].

### Production of antibodies and Western blot analysis

Two antibodies were raised against AdcA. New Zealand White rabbits were used to raise an anti-AdcA antibody (*r*Ab-AdcA) against a mixture of the two peptides ^14^AQESVDFVSSGFGN^27^ and ^523^CYPIATQGGNKYQSA^537^. The 108-day serum was purified on the peptides crosslinked to a Sepharose 4B column. Alternatively, *g*Ab-AdcA was obtained from Dunkin Hartley guinea pigs immunized against the purified H domain (see below) and purified from the 85-day serum on the recombinant domain. The specificity of *r*Ab-AdcA and *g*Ab AdcA was assessed by Western blot (dilution 1/500). In KAx-3 extracts separated by SDS-PAGE, a single band of AdcA of approximately the predicted size (65 kDa), and that is absent in the *adcA* knockout strain, was detected by both antibodies. The *r*Ab-AdcA antibody was not suitable for immunofluorescence.

### Protein purification

All recombinant proteins were expressed in *E. coli* BL21(DE3). Expression was induced by the addition of 1 mM IPTG at 37°C for 3 hr except when mentioned otherwise. The H domain and ArfA were purified by Ni^2+^-affinity chromatography, using the naturally occurring polyhistidine repeats or an added His_6_ tag respectively. Bacteria (corresponding to a 250 ml-culture) were thawed in buffer A (300 mM NaCl, 50 mM NaPi pH 8.0) containing 20 mM imidazole, protease inhibitors (1 µg/ml of aprotinin, leupeptin and pepstatin, 1 mM PMSF), 1 mg/ml lysozyme. After 15 min on ice, the bacterial suspension was pulse-sonicated for 3 min and soluble proteins cleared from the membranes by a 30 min centrifugation at 100000×*g*. The supernatant was mixed with Ni-NTA agarose beads (Qiagen, Courtaboeuf, France) for 1 hr at 4°C on a spinning wheel. After sequential washes in buffer A containing 50 to 80 mM imidazole, the H domain was eluted with buffer A containing 150 mM imidazole. Positive fractions were pooled and dialyzed overnight against buffer A adjusted to pH 7.5 for the H domain or 110 mM NaCl, 25 mM Hepes, 1 mM DTT, pH 7.5 for ArfA-His_6_. The dialyzed protein was cleared by full-speed centrifugation in an Eppendorf centrifuge for 15 min. MBP-FYVE expression was induced in LB medium supplemented with 10 µM ZnCl_2_. MBP-FYVE and MBP were purified on amylose resin (Ozyme, Saint-Quentin-en-Yvelines, France) as described previously [23]. GST-AdcA_C_ was expressed at 21°C (1 mM IPTG, 5 h). GST and GST-AdcA_C_ were purified by affinity on a glutathione-Sepharose column according to the manufacturer's instructions (GE Healthcare, Orsay, France). Protein concentrations were assayed with bicinchoninic acid using BSA as standard.

### Lipid overlay

Lipid dot-blot assays were performed according to the manufacturer's instructions (Echelon Biosciences). MBP and MBP-FYVE at a final concentration of 1 µg/ml were incubated with the PIP strips overnight at 4°C. After several washes, protein binding on the membrane was analyzed using an anti-MBP antibody.

### Pull-down

Affinity purified ArfA-His_6_ was preincubated for 1 hr at 4°C in 500 µl of a buffer containing 20 mM Tris, pH 7.5, 25 mM NaCl, 2 mM DTT, 2 mM EDTA, 2.5 mM MgCl_2_, 1 mM ATP, 0.2% Triton X-100 plus 100 µM GTPγS, GDPβS or no nucleotide. Equivalent amounts of GST or GST-AdcA_C_ bound to glutathione Sepharose beads (around 40 µl) were added to the mixture and further incubated overnight at 4°C. After several washes in the same buffer, Laemmli denaturating gel buffer was added directly to the beads and the proteins were analyzed by Western blot using the anti-Arf antibody.

### Microscopy

For immunofluorescence analysis, cells were allowed to adhere to glass coverslips (Labtek) for at least 20 min and fixed either in methanol at −20°C for 10 min or in 40 mM Mes-Na pH 6.5, 4% paraformaldehyde (PFA) for 30 min at room temperature. PFA-fixed cells were subsequently permeabilized with methanol (2 min at −20°C). Cells were then incubated first in PBS-0.5% BSA for 30 min and second with the indicated antibodies for 1 hr. After several washes, the cells were stained with corresponding fluorescent secondary antibodies for 1 hr. Observations were performed using a Zeiss Axiovert 200 M microscope. To probe the endocytic or phagocytic pathway, cells were let to internalize TRITC-dextran, Texas Red-zymosan or tetramethylrhodamine-conjugated *E. coli* for the indicated times. When mentioned, the cells were then washed in ice-cold 40 mM Mes-Na, pH 6.5 and suspended in fresh culture medium. Cells were observed directly or after fixation by confocal laser scanning microscopy, using a Leica TCS-SP2 operating system (Leica, Heidelberg, Germany) or by epifluorescence on a Zeiss Axiovert 200 M microscope (Zeiss, Le Pecq, France). When mentioned and in order to enhance resolution, optical sections were taken every 0.250 µm throughout the cell and digitally deconvolved using Axiovision software. Pictures were assembled using Adobe Photoshop or ACD Canvas software.

### Subcellular fractionation

*Dictyostelium* amoebae (2×10^7^ cells) were suspended in 300 µl of 20 mM Mes-Na, pH 6.5, 110 mM NaCl, 1 mM DTT plus protease inhibitors and vortexed with 0.3 g 0.17 mm-diameter glass beads. After addition of 400 µl of the same buffer, nuclei and unbroken cells were removed by 5 min of centrifugation at 1,000×*g* as described previously [24]. The membranes and cytosol were then separated by 30 min of centrifugation at 135,000×*g*. The pellet was resuspended in a volume of buffer equivalent to that of the soluble fraction. Equal volumes of membrane and soluble fractions were analyzed by denaturing polyacrylamide gel electrophoresis and Western blot analysis. To test the effect of carbonate treatment or pH variations, *Dictyostelium* amoebae were resuspended either in 100 mM Na_2_CO_3_, pH 11.0 or in 250 mM sucrose, 1 mM EDTA, 20 mM buffer at a given pH (Mes-Na, pH 6.0 or Hepes-Na pH 7.0 or Tris-HCl pH 7.5, pH 8.0 or pH 8.5) supplemented with protease inhibitors. Fractionation was then performed as described above.

## Results

### AdcA, an arrestin-domain containing protein in *Dictyostelium*

We have found 6 members of the arrestin subfamily that we named AdcA to -F for **a**rrestin-**d**omain **c**ontaining proteins A to -F in the *Dictyostelium* genome [15]. All *Dictyostelium* Adc proteins (Table S1) are significantly longer than canonical vertebrate arrestins, due to the presence of extra-domains besides the arrestin core. In addition to this arrestin core, five of the *Dictyostelium* Adc proteins exhibit at least one extra domain that is expected to mediate functional interactions with either lipids (C2 domain in AdcB and -C, FYVE domain in AdcA and -D) or proteins (SAM domain in AdcB and -C, MIT and LIM domains in AdcE) (Table S1).

**Figure 1:**
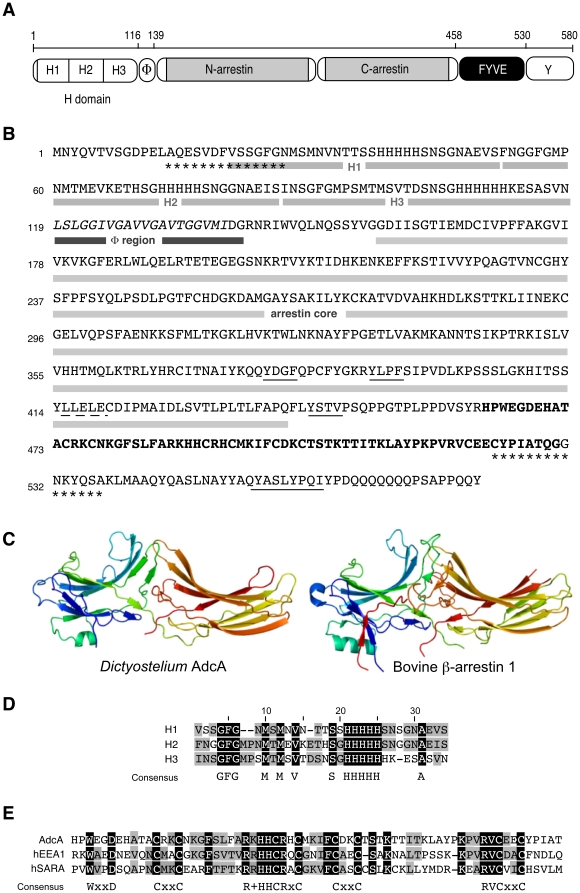
Characteristics of *Dictyostelium* AdcA. (**A**) Detailed domain structure of AdcA. Are indicated the H domain that contains the histidine-rich tri-repeat (H1-H2-H3), the hydrophobic region (Ф domain), the arrestin N- and C domains, the zinc-finger FYVE domain and the C-terminal extension rich in tyrosine residues (Y domain). (**B**) Protein sequence of AdcA. The limits of the H, Ф and arrestin domains are indicated by a thick gray or black underlining. Underlined AdcA residues correspond to putative clathrin- (dotted line) and adaptin- (continuous line) binding motifs. Stars indicate the two peptides used to produce the rAb-AdcA antibody. (**C**) Homology modeling of the *Dictyostelium* AdcA core (amino acids 140–460). Residues are shown in ribbon format, with the color code running from blue (N-terminal) to red (C-terminal) along the polypeptide chain. The 2WTR 3D-structure corresponding to bovine β-arrestin 1 is shown for visual comparison with AdcA's modeled structure. (**D**) Alignment of the histidine-rich repeats of the H domain. Identical amino acids in the three repeats H1, H2, and H3 are shaded in black. Conservative changes or residues conserved in only two repeats are shaded in gray. Dashes indicate gaps. (**E**) Alignment of FYVE domains present in *Dictyostelium* AdcA, human EEA1 and human SARA proteins. Identical amino acids in the three sequences are shaded in black. Conservative changes or residues conserved in only two sequences are shaded in gray. Dashes indicate gaps and (+) stands for lysine or arginine.

In this report, we chose to focus on the protein AdcA from *D. discoideum* (Figure 1A–B). Arrestin-domain containing proteins extended with a FYVE domain were also found in *D. purpureum*, *Polysphondylium pallidum*, *Entamoeba histolytica and E. dispar*, other members of the Conosea group within the Amoebozoa supergroup to which belongs *D. discoideum*. Interestingly, a FYVE domain-containing arrestin structured as AdcA was also found in two species of a picoeukaryote, *Micromonas pusilla* CCMP1545 and *Micromonas* sp. RCC229 with expect values of 1×e^−24^ and 8×e^−24^, respectively (Figure S1). *Micromonas* belongs to the class of marine phytoplankton Prasinophyceae, ancient members of the green lineage that gave rise to higher plants. No arrestin-domain protein harbouring a FYVE domain was however found in green plants.

Homology modeling of the arrestin core of *Dictyostelium* AdcA was attempted using the CPH software available online at http://www.cbs.dtu.dk/services/CPHmodels/ on the basis of a PSI-Blast expect value of 1×e^−31^ with bovine β-arrestin 1. The predicted 3D-structure obtained for AdcA's arrestin core is shown beside the structure of β-arrestin 1 (2WTR) for visual comparison (Figure 1C). This result is a strong indication that *Dictyostelium* AdcA is organized similarly to mammalian arrestins, despite its limited sequence homology. Thus, it may function in similar conserved processes and act as a multifunctional scaffold in protein complexes linking membrane receptors to intracellular pathways.

As mentioned above, AdcA harbors extensions on both sides of the arrestin core (Figure 1A). The arrestin core is N-terminally stretched out by a 23-aa hydrophobic sequence (^119^Ф^138^). Analysis of this Ф stretch with the HeliQuest software (http://heliquest.ipmc.cnrs.fr) predicted an amphipathic α-helix despite the presence of glycines known as helix breakers, with a hydrophobic face (AIGVLVVVV) and polar uncharged residues on the other face of the helix suggesting a possible interaction with membranes or hydrophobic regions in proteins [25]. An additional feature within the N-terminal region was detected by visual inspection. As shown in Figure 1D, AdcA presents a triple ∼30-aa repeat, each including a poly-histidine cluster of 5 to 6 contiguous histidine residues (H domain) and sharing almost 80% homology. A similar histidine-rich region is found in the AdcA isoform from *D. purpureum* as a quadruple repeat, but is absent in the *P. pallidum* isoform (Figure S1). No homolog was found elsewhere for this so far undescribed arrangement. A canonical FYVE domain (PF01363) and a tyrosine rich-region (Y domain) extend the protein on the C-terminal side. The FYVE domain harbors the consensus eight zinc-coordinating cysteine residues and inositol phospholipid binding domain (signature WxxD, R(R/K)HHCR and RVC) and shares 42% identity and 63% similarity over a 70 amino acid stretch with that of EEA1 (Figure 1E). AdcA contains a potential canonical clathrin box sequence (^415^LLELE^419^) in the arrestin core and several putative tyrosine-based sorting signals likely to interact with the µ subunit of adaptor proteins (Figure 1B). In its C-terminal tyrosine rich domain, AdcA also carries 2 SxP sites within 2 PSxPP sequences and a YPxL/I site. Such sites are described as binding sites for ESCRT subunits or ESCRT-associated proteins [26], [27].

### AdcA developmental regulation

Under nutritive conditions, *D. discoideum* grows as a unicellular organism. Starvation triggers a 24 hr-long developmental program leading to the formation of multicellular fruiting bodies containing spores [28]. Expression of AdcA throughout the KAx-3 cell developmental program (referred to as the wild-type thereafter) was followed by Western blot using the *r*Ab-AdcA polyclonal antibody. As shown in Figure S2, AdcA expression is highest during vegetative growth and rapidly decreases as cells enter development. The disappearance of AdcA at the onset of development is likely due to a rapid degradation of the protein coupled to a reduction in the rate of transcription. Such an expression time course is suggestive of a role of the protein in the vegetative stage and/or in the transition from growth to development.

### AdcA is associated with the endocytic pathway

**Figure 2:**
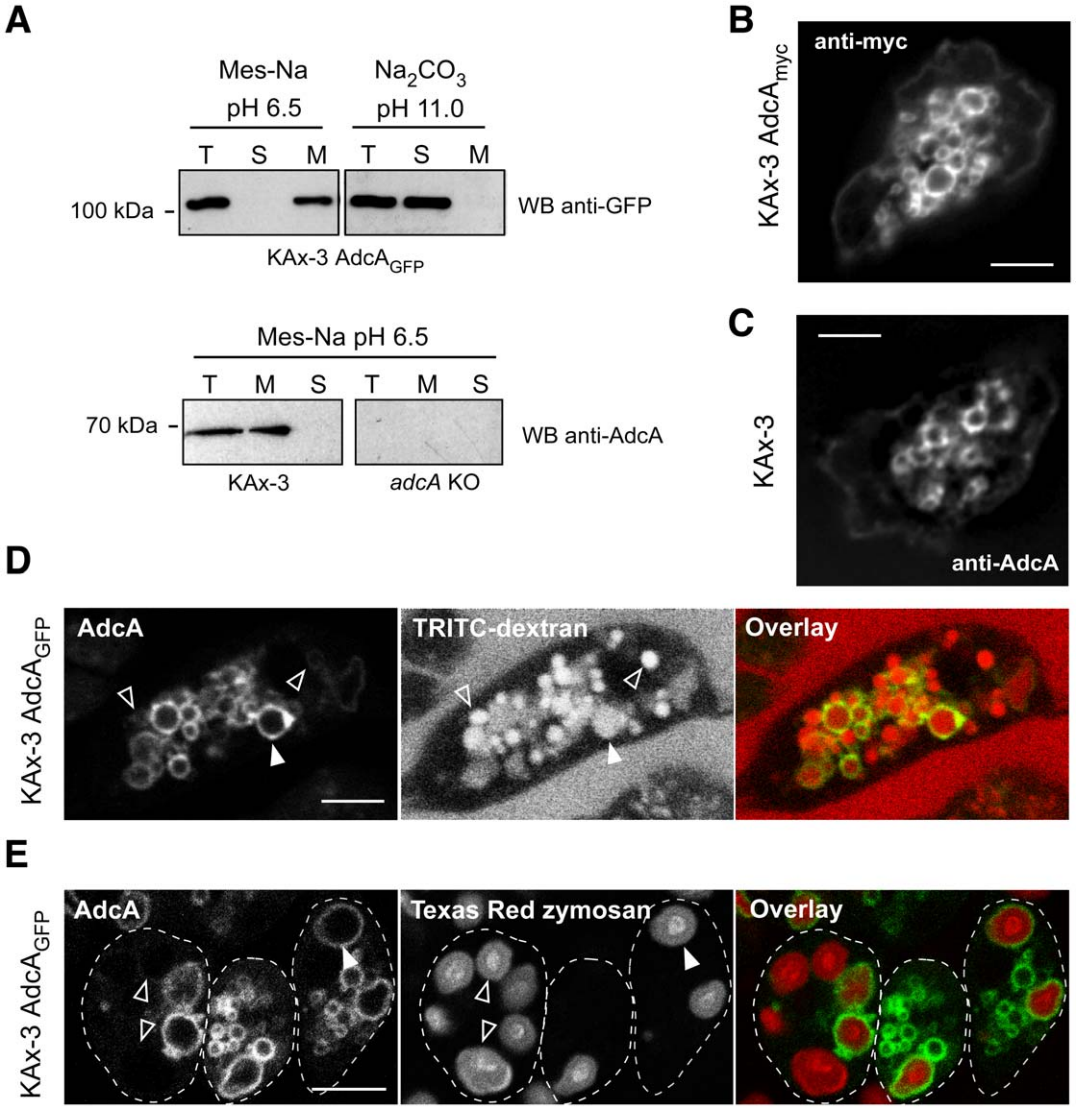
AdcA is docked to endocytic compartments. (**A**) Subcellular fractionation. KAx-3 and KAx-3 AdcA_GFP_ cells were broken in different buffers (Mes-Na pH 6.5 or Na-carbonate pH 11) as mentioned in Materials and Methods. The postnuclear supernatant was spun by high-speed centrifugation (30 min, 135,000×*g*, TL100 Beckman) to separate membranes and soluble fractions. Samples were analyzed by Western blot using either gAb-AdcA or anti-GFP antibodies. The *adcA* null strain was used as a control. T, total before centrifugation; S, soluble fraction; M, membranes. (**B**), (**C**) AdcA is present on endocytic vesicles. KAx-3 and KAx-3 expressing AdcA_myc_ were fixed in methanol and processed for immunofluorescence with an anti-myc antibody (**B**) or the gAb-AdcA antibody (**C**). The scale bar represents 3 µm. (**D**) Cells expressing AdcA_GFP_ were let to internalize the fluid-phase marker TRITC-dextran for 2 hr. Live imaging was then performed on a Leica TCS-SP2 confocal microscope. Panels illustrate the location of AdcA_GFP_, TRITC-dextran and the overlay in false colors of both fluorescences. The full arrowhead points to a vesicle positive for both TRITC-dextran and AdcA_GFP_. Open arrowheads point to vesicles positive for TRITC-dextran but negative for AdcA_GFP_. (**E**) Cells expressing AdcA_GFP_ were let to internalize Texas Red-labeled zymosan BioParticles® for 2 hr. Live imaging was then performed on a Leica TCS-SP2 confocal microscope. Panels illustrate the location of AdcA_GFP_, Texas Red-labeled zymosan and the overlay in false colors of both fluorescences. The full arrowhead points to a vesicle positive for both Texas Red-labeled zymosan BioParticles® and AdcA_GFP_. Open arrowheads point to vesicles positive for BioParticles® but negative for AdcA_GFP_. A dashed line delineates cell outlines. The scale bar in (**D**) and (**E**) represents 4 µm.

Because of its temporal expression profile, we focused the analysis of AdcA distribution on vegetative cells. Subcellular fractionation assays and microscopy approaches were used. We engineered tagged full-length Adc (AdcA_myc_, AdcA_GFP_, _RFP_AdcA) and overexpressed the various proteins in the KAx-3 strain under a constitutive promoter. In fractionation assays performed in 20 mM Mes-Na pH 6.5, AdcA_GFP_ distributed within the particulate fraction after centrifugation of the post-nuclear supernatant at 100000×*g* (Figure 2A). A similar distribution was obtained for the endogenous AdcA (Figure 2A). Because of the possibility that the hydrophobic stretch may organize as a membrane-spanning segment, we tested the effect of a treatment with 0.1 M Na-carbonate, pH 11.0. Such treatment fully released AdcA_GFP_ into the soluble fraction (Figure 2A), arguing against an intrinsic character of AdcA.

To determine the subcellular localization of AdcA, the distribution of the myc-tagged AdcA was observed by immunofluorescence. In fixed cells, AdcA_myc_ strongly decorated the rim of vesicular structures up to 1–2 µm in diameter, reminiscent of the compartments of the macropinocytic pathway. A faint fluorescence was also associated with the plasma membrane (Figure 2B). A similar staining was obtained with AdcA_GFP_- and _RFP_AdcA-expressing cells (see below). On parental cells, the *g*Ab-AdcA antibody confirmed the vesicular localization of AdcA (Figure 2C). This antibody could not be used to validate the localization of AdcA at the plasma membrane as it decorated non-specifically the plasma membrane of *adcA* knockout cells. To determine the nature of the AdcA-positive vesicles, AdcA_GFP_-expressing cells were incubated for 2 hr with the fluid-phase marker tetramethylrhodamine isothiocyanate-dextran (TRITC-dextran) and observed by confocal laser scanning microscopy. TRITC-dextran is taken up by *Dictyostelium* cells by macropinocytosis and selectively labels the compartments of the pathway. Vesicular AdcA_GFP_ located exclusively on TRITC-dextran containing compartments (Figure 2D, full arrowheads). Noteworthy, some TRITC-dextran-positive vesicles were devoid of AdcA_GFP_ (Figure 2D, empty arrowheads) indicating that AdcA associates with a specific subset of macropinocytic compartments. As shown in Figure 2B the distribution of AdcA along the rim of the vesicles was irregular with local thickenings or gaps along the membrane. The meaning for this heterogeneity is currently unknown, but might correspond to membrane areas involved in fusion-fission processes.

To assess whether AdcA is a specific marker for the macropinocytic versus phagocytic pathway, cells expressing AdcA_GFP_ were fed with Texas Red-labeled zymosan BioParticles® for 2 hr. AdcA_GFP_ located on phagosomes containing zymosan Bioparticles® (Figure 2E, full arrowheads). As observed above for macropinosomes, some phagosomes could be observed that carry less or no AdcA_GFP_ (Figure 2E, empty arrowheads). Similar results were obtained with AdcA_myc_ expressing cells fed with tetramethylrhodamine-conjugated *E. coli* (Figure S3).

### AdcA is restricted to early endosomes

**Figure 3:**
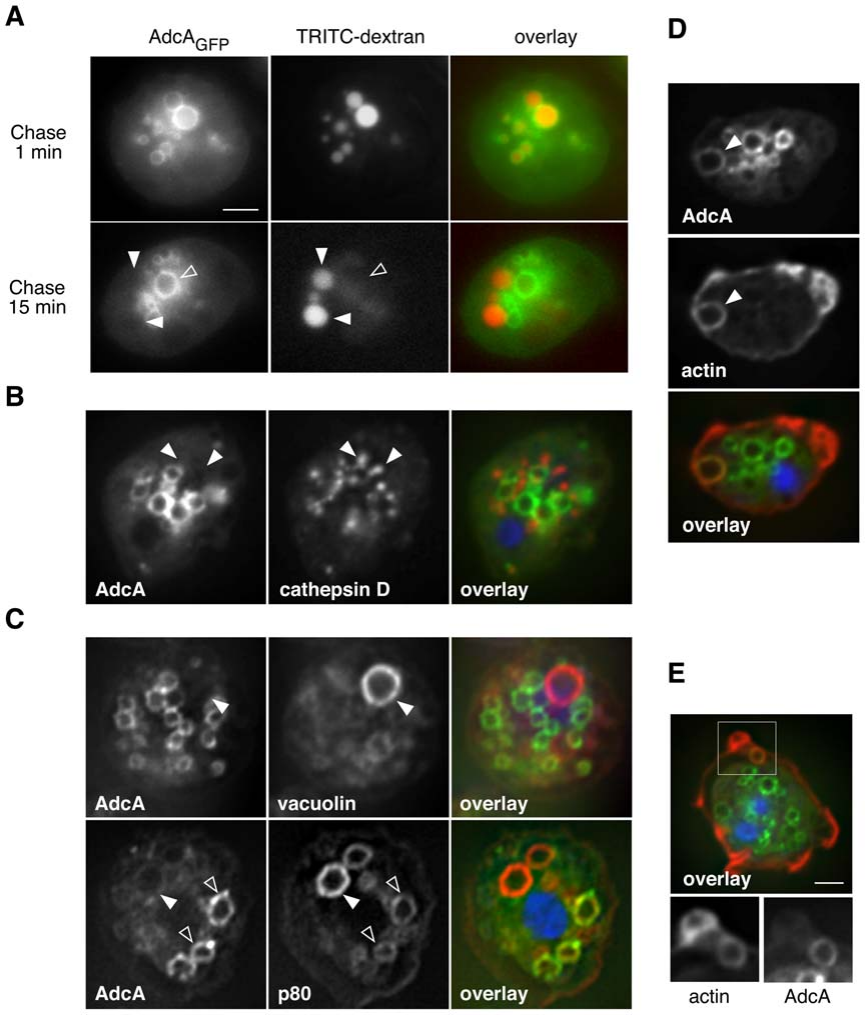
AdcA_GFP_ is present on early endocytic compartments. (**A**) Cells expressing AdcA_GFP_ were pulsed with TRITC-dextran for 5 min. After several washes to remove the external marker, cells were resuspended in fresh culture medium and visualized after 1 min chase (top) or 15 min chase (bottom) on a Zeiss Axiovert 200 M microscope. During the chase period, cells accumulated compartments positive for TRITC-dextran but devoid of AdcA_GFP_ (full arrowheads). New endosomes formed during the chase period were positive for AdcA and negative for TRITC-dextran (empty arrowhead). (**B–E**) Cells expressing AdcA_GFP_ were fixed in methanol and processed for immunofluorescence with anti-cathepsin D (**B**), anti-vacuolin (**C**, top), anti-p80 (**C**, bottom) and anti-actin (**D**, **E**) antibodies as well as DAPI to stain the nucleus. In panel (**C**), a strongly p80-positive, AdcA-negative post-lysosome is indicated by a full arrowhead. p80-positive, AdcA-positive endosomes are indicated by empty arrowheads. Panel (**E**) illustrates the concentration difference of AdcA on a macropinocytic cup and an early macropinosome identified by actin staining. For panels (**B**), (**C**), (**D**) and (**E**), in order to enhance resolution, optical sections were taken every 0.250 µm throughout the cell and digitally deconvolved using Zeiss Axiovision software. A median z section is shown. The scale bar represents 2 µm.

In *Dictyostelium*, the fluid phase transits sequentially through distinct compartments (endosomes, lysosomes and post-lysosomes) defined on the basis of their internal pH and the presence of specific markers and which are temporally well-defined along the pathway [29]–[31]. To specify the age and the nature of the AdcA_GFP_-positive compartments, cells were subjected to pulse-chase experiments (Figure 3A). After a 5 min pulse of TRITC-dextran and a 1 min chase, conditions that are reported to label endosomes [30], the compartments loaded with the fluid-phase marker, and only these, were positive for AdcA_GFP_. As the chase period increased (≥15 min), the fluid-phase marker reached first lysosomal and later post-lysosomal compartments. These TRITC-dextran loaded vesicles did not carry AdcA_GFP_. However, AdcA_GFP_ was present on TRITC-dextran negative endosomes that necessarily formed during the chase period. This observation indicates an enrichment of AdcA_GFP_ on early endosomes and its retrieval prior to the later compartments of the endocytic pathway (Figure 3A). This point was confirmed by immunofluorescence studies, using antibodies against marker proteins of lysosomes or post-lysosomes. The anti-cathepsin D antibody was used to decorate the lysosomes. As shown in Figure 3B, AdcA_GFP_ is excluded from the typical lysosomal punctuate staining. The anti-vacuolin and anti-p80 (H161) antibodies were used to label the post-lysosomes [16], [17]. Contrary to vacuolin that is specific for post-lysosomes, the membrane protein p80 is present throughout the endocytic pathway with a strong accumulation on post-lysosomal vacuoles. In fixed cells expressing AdcA_GFP_, anti-vacuolin and anti-p80 strongly delineate large size post-lysosomes. These compartments carry no AdcA_GFP_ (Figure 3C, full arrowheads). These observations are in agreement with the pulse-chase data and establish that AdcA_GFP_ is restricted to the early endocytic compartments.

To precise the age of the endosome at the time of AdcA_GFP_ enrichment, we labeled cells with an anti-actin antibody. Macropinocytosis depends on the actin cytoskeleton as it contributes to the formation of membrane protrusions that allow fluid uptake in *Dictyostelium* [32], [33]. The actin coat associated with the macropinocytic cup is removed from the nascent macropinosome within the minute following its formation. In growing cells expressing AdcA_GFP_, newly formed macropinosomes, which can be identified by the anti-actin antibody are also positive for AdcA_GFP_ while only a faint staining is visible on macropinocytic cups (Figure 3D–E). The protein AdcA_GFP_ is therefore highly enriched just after closure of the nascent macropinosome. AdcA_GFP_ is then retrieved from the macropinosome as it matures and acquires lysosome-specific characteristics.

### The FYVE domain is essential but not sufficient for the endosomal association of AdcA

**Figure 4:**
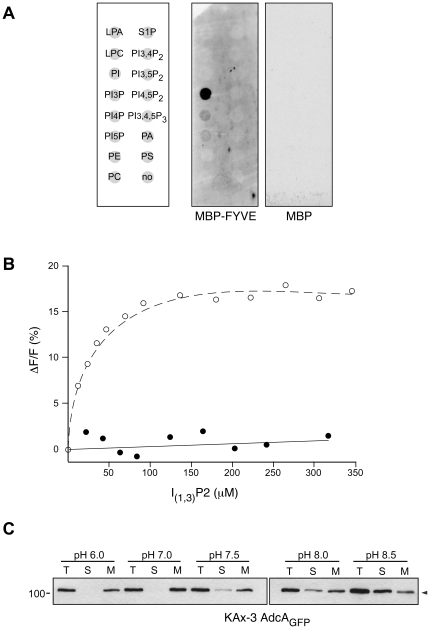
AdcA harbors a functional FYVE domain. (**A**) Lipid overlay assay. AdcA FYVE domain was expressed as a MBP-fusion protein and tested for its binding to lipids immobilized on a membrane. MBP alone was used as a control. LPA, lysophosphatidic acid; LPC, lysophosphocholine; PI, phosphatidylinositol; PI3P, phosphatidylinositol 3-phosphate; PI4P, phosphatidylinositol 4-phosphate; PI5P, phosphatidylinositol 5-phosphate; PE, phosphatidylethanolamine; PC, phosphatidylcholine; S1P, sphingosine 1-phosphate; PI3,4P_2_, phosphatidylinositol 3,4-bisphosphate; PI3,5P_2_, phosphatidylinositol 3,5-bisphosphate; PI4,5P_2_, phosphatidylinositol 4,5-bisphosphate; PI3,4,5P_3_, phosphatidylinositol 3,4,5-trisphosphate; PA, phosphatidic acid; PS, phosphatidylserine; no, blank. (**B**) Binding of Ins(1,3)P_2_. Variations of tryptophan intrinsic fluorescence (λ_ex_ 290 nm) of MBP-FYVE (o) and MBP (•) were measured between 300 and 400 nm in response to addition of Ins(1,3)P_2_, a soluble analog of PI(3)P. Variations were expressed as ΔF/F_0_ in percent. (**C**) AdcA membrane association is pH dependent. Cells expressing AdcA_GFP_ were broken in different buffers at different pHs and treated as in panel (**A**).

As stated above, AdcA diverges from genuine arrestins because of its N- and C-terminal extensions organized as domains among which a FYVE domain with its characteristic consensus signature. Most FYVE domains, such as that of EEA1, SARA and Hrs are known to bind a specific phosphoinositide, the PI(3)P with high affinity, thereby allowing or contributing to the recruitment of the proteins on PI(3)P-enriched organelles including endosomes. Lipid-binding specificity of AdcA FYVE domain was assessed in a lipid overlay assay using ECHELON membranes spotted with various phospholipids and the recombinant FYVE domain (amino acids 458–530) N-terminally fused to the maltose-binding protein MBP (MBP-FYVE). The MBP protein alone was used as a control in the assay. The only strong binding activity of MBP-FYVE was observed for the monophosphoinositide PI(3)P (Figure 4A). Much weaker interactions were observed with two other phosphoinositides, PI(4)P and PI(5)P. The binding affinity of AdcA FYVE domain for the PI(3)P head group was measured by monitoring intrinsic tryptophan fluorescence variations of MBP-FYVE as a function of added Ins(1,3)P_2_, a soluble analog of PI(3)P (Figure 4B). The affinity of MBP-FYVE deduced from the Michaelian fit of the plot was around 20 µM. This value compares with published affinities (25–140 µM) of EEA1 and SARA for Ins(1,3)P_2_ [34]. The affinity of AdcA FYVE for its physiological ligand PI(3)P is expected to be better by a factor of 100–1000 as measured for other FYVE domains [35].

As a first approach to test a role for PI(3)P recognition in AdcA endosomal targeting, cells were treated with the PI3K inhibitor LY294002 (25 µM). In agreement with the literature, LY294002 was found to act very rapidly. Within 5 min following addition of the drug and with the exception of very occasional cells that maintained a faint staining of their macropinosomes, the vast majority of the LY294002-treated cells displayed a cytosolic localisation of AdcA_GFP_ with a complete loss of the vesicular distribution that was observed in DMSO-treated control cells (Figure S4). Tiny spots were visible in the cytoplasm, possibly corresponding to protein aggregates. In the light of the lipid overlay experiment, this observation supports the idea that AdcA binding to PI(3)P contributes to its endosomal recruitment.

Binding of FYVE domains to PI(3)P-enriched membranes is regulated by a histidine switch that involves the two histidines of their highly conserved R(R/K)**HH**CR motif. As a consequence, binding is strongly pH-dependent. In HeLa cells, increasing cytosolic pH is sufficient to prevent EEA1 binding on endosomal membranes [36]. To test the contribution of the FYVE domain to the endosomal association of AdcA, we first examined the partitioning of AdcA_GFP_ between soluble and membrane fractions when cells were broken at different pH values between 6.0 and 8.5. As shown above, at a pH below 7.0, the protein was retained in the pelletable fraction (Figures 4C and 2A). As the pH of the incubation buffer reached higher values, AdcA_GFP_ distributed progressively into the soluble fraction. At pH 8.5, AdcA_GFP_ partitioned equally between both soluble and membrane fractions (Figure 4C). This behavior fully reflects that of canonical FYVE domains with respect to pH-dependency [35] and points to AdcA FYVE domain as being the motif responsible for the targeting of AdcA to PI(3)P-containing membranes.

**Figure 5:**
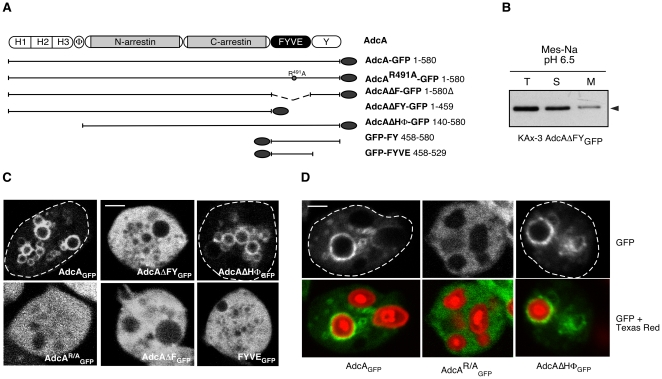
Role of AdcA subdomains in its endosomal location. (**A**) Deletion constructs. To test the contribution of AdcA subdomains to the protein subcellular location, various truncated constructs were generated, tagged with the GFP at the N- or C-terminus and expressed in KAx-3 cells under *act15* promoter. Numbers correspond to the constructs borders. (**B**) The distribution of AdcAΔFY_GFP_ after fractionation was analyzed by Western Blot as described in Figure 2. (**C**) The location of the GFP-tagged truncated proteins was observed in live cells by confocal microscopy to evaluate the effect of the truncation on the endosomal docking during macropinocytosis. (**D**) Cells expressing the mentioned constructs were let to internalize Texas Red-labeled zymosan BioParticles® for 2 hr prior observation. The upper panel illustrates the subcellular location of the AdcA_GFP_ constructs and the lower panel corresponds to the overlay of green (AdcA constructs) and red (phagocytic probe) fluorescences. The scale bar represents 2 µm.

To assess this role in more detail, we generated several deletions removing specific domains of the protein (Figure 5A). Deletion of the C-terminal region of AdcA (AdcAΔFY_GFP_, amino acids 1–459) had a dramatic effect on the location of the protein. In subcellular fractionation assays, more than 80% of the AdcAΔFY_GFP_ partitioned in the soluble fraction under conditions (pH 6.5) where AdcA_GFP_ is fully membrane bound (Figure 5B). When observed by confocal microscopy, these same cells displayed a diffuse cytoplasmic staining with occasional aggregates within the cytosol that could account for the remaining portion of the protein found in the pelletable fraction (Figure 5C). No vesicular staining remained visible, which supports the hypothesis of a role for the AdcA C-terminal domain in endosomal targeting. The strong cytosolic fluorescence precluded the observation of plasma membrane association of AdcA. Similar results were obtained with a specific deletion of the FYVE domain, AdcAΔF_GFP_ (Figure 5C). However, to exclude the possibility of an indirect effect of the deletions on AdcA conformation, we introduced a single point-mutation R^491^A in the FYVE domain (AdcA^R/A^), based on previous studies that demonstrated that this conversion abrogates PI(3)P binding without affecting overall conformation of the domain [37]. The R^491^A mutant lost its ability to bind endosomes showing unequivocally the necessity of the domain for endosomal recruitment (Figure 5C). Similar results were obtained with respect to recruitment of AdcA on phagosomes (Figure 5D). To establish whether the FYVE module was sufficient for association to the endocytic compartments, we created a series of truncated mutants in the upstream and downstream domains of AdcA (_GFP_FYVE-Y or FYVE-Y_GFP_, _GFP_FYVE). The GFP-tagged FYVE domain, by itself (Figure 5C) or associated with the Y domain (not shown), failed to target to the endosomes. In the case of mammalian EEA1 or Hrs FYVE domains, endosomal targeting was achieved when the respective FYVE domains were expressed in tandem [38]. This approach, applied to the AdcA FYVE domain, did not allow its vesicular recruitment. Several hypotheses can be proposed to explain this observation: (1) the FYVE domain is sufficient for endosomal membrane binding but the constructs failed to adopt the appropriate conformation to the bind the endosomal membrane, (2) the FYVE domain is not sufficient and another domain of the protein contributes to binding together with the FYVE domain.

The AdcA N-terminal extremity includes a histidine-rich triple-repeat (H domain) and a hydrophobic stretch (Φ domain) whose functions are unknown. Because of the hydrophobic nature of this extremity, we wondered whether it might contribute to membrane anchoring together with the FYVE domain. A deletion removing both the H- and Ф domains (AdcAΔHФ) maintained its ability to target GFP to macropinosomes and phagosomes (Figure 5C–D). To exclude the possibility that AdcAΔHФ was recruited on endosomes via oligomerization with the endogenous AdcA, localization of AdcAΔHФ was also assessed in the *adcA* knockout strain (Figure S5A). Even in the absence of endogenous AdcA, AdcAΔHФ was associated with macropinosomes (Figure S5B), indicating that the HФ extension is dispensable for endosomal targeting. Along the same line, deletions removing the arrestin core (AdcAΔNC) or the Y domain (AdcAΔY) were generated but in both cases, no expression of the truncated proteins was detectable, precluding functional conclusions. Whether these other domains or a membrane-associated partner tighten the interaction with endosomes remains therefore to be established.

### AdcA interacts with GDP-bound ArfA

**Figure 6:**
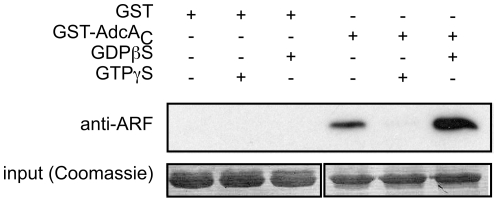
The C domain of AdcA arrestin core interacts with Dd-ArfA *in vitro*. AdcA_C_ was expressed and purified as a GST-fusion protein. ArfA was tagged with a C-terminal His_6_ tag and purified on a Ni-NTA column. GST-Adc_C_ and GST were kept bound to the glutathione Sepharose beads and incubated overnight at 4°C with ArfA in the presence of 100 µM GDPβS, GTPγS or no extra nucleotide. After several washes in incubation buffer, beads were resuspended in Laemmli denaturating gel buffer and proteins were analyzed by Western blot for the presence of ArfA.

In mammals, β-arrestins function together with several proteins of the endocytic machinery including AP-2 adaptin, clathrin, NSF and the ADP-ribosylation factor Arf6 and its exchange factor ARNO [39]. ArfA is the only member of the Arf protein family (Arf1-6 in mammals) present in the *Dictyostelium* genome [40]. We examined the possibility of an interaction between the AdcA arrestin core and ArfA in pull-down assays using purified bacterially expressed proteins. As AdcA_NC_ was insoluble as a fusion protein with GST, we limited our test to the C-terminal domain of the AdcA arrestin core (GST-AdcA_C_). ArfA was purified using the polyhistidine tag added to its C-terminal extremity. ArfA-His_6_ bound to GST-AdcA_C_ and, more interestingly, binding was dependent on the nature of the nucleotide present on the small G-protein (Figure 6). Indeed, interaction occurred in the presence of GDPβS as well as in the absence of extra nucleotides. Conversely, addition of GTPγS that stabilizes/favors a GTP-bound form of Arf proteins markedly impaired interaction (Figure 6). Our results therefore indicate that ArfA directly binds to the arrestin C-domain of AdcA and that the binding conformation is the GDP-loaded form.

**Figure 7:**
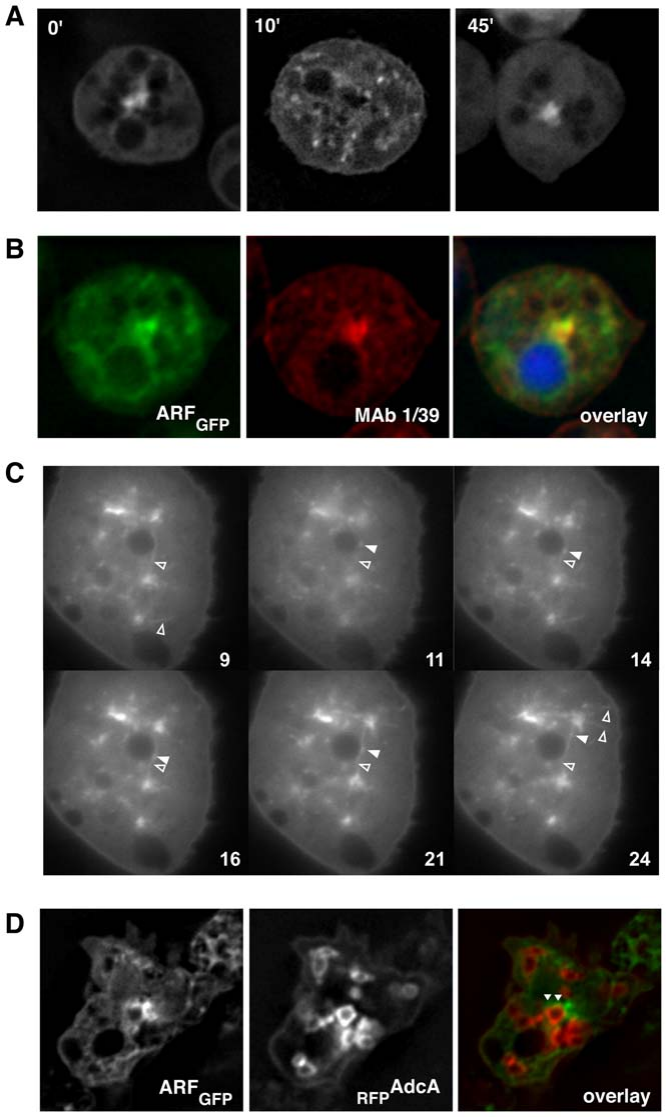
Subcellular localization of ArfA. (**A**) KAx-3 cells expressing ArfA_GFP_ were let to adhere in Labtek chambers. DMSO was added (t = 0) at a final concentration of 5% and cells were observed on a Zeiss Axiovert 200 M microscope immediately or after 10 min or 45 min of incubation. (**B**) KAx-3 cells expressing ArfA_GFP_ were fixed in 4% PFA, permeabilized in methanol and stained with the monoclonal antibody 1/39 described to label the Golgi apparatus [55]. The overlay panel correspond to the overlay of the green and red fluorescences in addition to the nuclear DAPI staining. (**C**) Cells expressing both ArfA_GFP_ and _RFP_AdcA were fixed and observed. The arrow indicates an AdcA positive vesicle in close proximity with the Golgi apparatus. (**D**) Live cells expressing ArfA_GFP_ were observed by time-lapse microscopy. Individual pictures corresponding to the indicated frames (obtained every 0.25 sec) were extracted from the movie (Movie S1). ArfA-positive tubules are indicated by empty arrowheads. The full arrowhead in frames 11–24 shows an ArfA-positive vesicle associated with an ArfA-positive tubule, first moving away from the Golgi apparatus (centrifugal direction) and finally reversing its movement (centripetal direction).

The interaction of AdcA with ArfA prompted us to investigate the subcellular localization of ArfA using KAx-3 cells expressing a C-terminally GFP-tagged ArfA (ArfA_GFP_). As shown in Figure 7A, ArfA_GFP_ was found in the cytosol, at the plasma membrane but mostly localized as a patch in the perinuclear region, suggesting an association with the Golgi apparatus confirmed by immunostaining with the Golgi marker antibody 1/39 (Figure 7A–B). As expected for a Golgi association [41], the perinuclear patch was rapidly disrupted by a treatment with 5% DMSO and reconstituted in the next 45-min (Figure 7A). Observation of living cells by time-lapse microscopy also showed the presence of ArfA_GFP_ on rapidly moving structures including vesicles and tubules (Movie S1). ArfA_GFP_-positive vesicles were seen moving away from and towards the Golgi apparatus (Figure 7C). This observation is consistent with a role for ArfA in trafficking events linking the Golgi apparatus to organelles such as the endosomes, as reported for mammalian cells. Though ArfA was found by proteomic analysis on phagosomes [42] and on magnetically purified endosomes (our unpublished work), no ArfA_GFP_ staining was convincingly seen associated with the phagosomes or macropinosomes (Figure 7D), possibly due to transient interactions. However, some ArfA_GFP_ spots were detected in close vicinity of AdcA-positive endosomes (Figure 7D, arrowheads).

## Discussion

### AdcA, an arrestin-related protein on early endosomes

β-arrestins have recently emerged as the indispensable scaffolding protein in a wide range of cellular processes, as they interact with an exponentially growing list of partners and targets at multiple subcellular sites of action. Their structure is organized as a bilobal β-sheet sandwich. Novel proteins with that same arrestin domain or fold have recently been described (Vps26, yeast ARTs, human ADCs) and they form, together with visual arrestins and β-arrestins, the arrestin clan [43]. The amoebal protein AdcA belongs to this superfamily. It contains the two N- and C-domains of arrestins as defined by the PFAM database and modeling of its arrestin core onto the structure of mammalian β-arrestin 1 indicates that despite poor sequence conservation with canonical arrestins, AdcA adopts a similar fold. When examined in the light of Alvarez classification into α- and β-arrestins classes [43], AdcA qualifies as a β-arrestin because of the absence of any PY sequence and the presence of an α-helix I in its modeled structure.

However, on the contrary to mammalian β-arrestins, AdcA is massively enriched on the early compartments of the macropinocytic and phagocytic pathways, with traces on the plasma membrane. Enrichment occurs just after the closure of the endocytic vesicles and their release from the plasma membrane and this distribution is highly dependent on a functional FYVE-type zinc-finger domain that extends the protein on the C-terminal part. Such domain is absent from canonical β-arrestins and may provide a way to restrict AdcA function to a specific site. So far, it is unknown whether the recruitment of AdcA on macropinosomal and phagosomal membranes follows some receptor/target activation, as it is the case for visual- and β-arrestin 1 and 2 recruitment at the plasma membrane. Because of the FYVE domain, AdcA could be docked on PI(3)P-enriched macropinosomes in a constitutive manner and thereon encounter the appropriate activation stimulus. Because of its 150 aa-long extension, which includes the FYVE domain and a tyrosine-rich region, AdcA C-terminal tail completely differs from that of β-arrestins. In line with this difference, no obvious polar core of buried salt bridges between the N-, the C-domain and the C-terminal tail was found in the modeled structure of AdcA. It is therefore likely that AdcA uses a mode of activation different from that of canonical arrestins.

AdcA is the first exemple of a FYVE domain-containing protein described in details in *Dictyostelium*. Both phagocytosis and macropinocytosis in *Dictyostelium* are dependent on PI(3)P synthesis, but no reporter for this phosphoinositide has been used to precisely establish its distribution along the endocytic pathway [44], [45]. We show here that PI(3)P, as demonstrated by the use of AdcA as a reporter, is present on incoming vesicles, once the membrane is sealed. This situation reproduces that observed in macrophages where PI(3)P is accumulated on phagosomes upon engagement of Fcγ receptors, just after closure of the vesicles [46], [47]. When compared to other phosphoinositides in *Dictyostelium*, the spatiotemporal dynamics of PI(3)P (as probed by AdcA) compare to that of PI(3,4)P_2_ round the closed phagosome/macropinosome whereas PI(3,4,5)P_3_ levels increase as soon as the phagocytic cup is initiated and rapidly decrease after closure of the vesicle, most probably by hydrolysis into PI(3,4)P_2_ [44]. Though the dynamics of PI(3,4)P_2_ and PI(3)P are compatible with PI(3)P deriving from PI(3,4)P_2_ hydrolysis, it is unlikely as no phosphatidyl inositol 4-phosphatase is reported in the curated *Dictyostelium* genome. PI(3)P is therefore expected to be generated by class 3 phosphatidyl inositol 3-kinases and PikE, the homolog of *S. cerevisiae* Vps34, could be the kinase responsible of its synthesis [48]. A functional FYVE domain is crucial for endosomal targeting of AdcA, but it is not sufficient, on its own or as a dimer to drive recruitment on the endosomes. Efficient membrane binding may rely on other domains allowing oligomerization of the protein and thereby increasing avidity for membrane-embedded PI(3)P or direct interaction with membrane-associated partners. Obviously, the N-terminal extension does not fulfill this function, as its deletion has no effect on the endosomal localization of AdcA.

### ArfA, a partner of AdcA

In mammals, β-arrestin 2 was shown to interact directly with the ADP-ribosylation factor ARF6, a molecular switch involved in vesicle trafficking and actin network remodeling. ARF6 belongs to the Ras-related ARF family that comprises 5 other members (ARF1–5) besides ARF-like, ARF-related and Sar proteins. Our data indicate that the GDP-bound form of ArfA, the only member of the ARF family in *Dictyostelium*, and only this nucleotide-bound form binds directly to the C-domain of AdcA *in vitro*. A similar regulation by the nature of the nucleotide was observed for β-arrestin 2/ARF6 interaction [49]. As expected from its close homology to ARF1, ArfA is primarily present on the Golgi apparatus, with a small fraction distributed between the cytosol and the plasma membrane. The close proximity of the bulk Golgi-located ArfA with AdcA-positive vesicles, the presence of ArfA on tubules and vesicles in connection with the Golgi apparatus together with its identification in proteomic analyses of purified phagosomes [42] and macropinosomes (LA, DG and GK, unpublished data) supports a role for ArfA in vesicular trafficking between the endocytic pathway and the Golgi network. Mammalian and trypanosomal ARF1s have no absolute Golgi location neither and localize also on punctuate endosomal structures [50] or at the plasma membrane respectively in addition to their soluble distribution [51]. A function of ARF1 during the extension and closure steps of phagosomes, after the phagosomal cup formation initiated by ARF6, has also been described [52]. In *Dictyostelium*, the functional link between AdcA and ArfA is not clear yet. The interaction between AdcA and ArfA may be transitory making it particularly difficult to observe a convincing co-localization. AdcA and ArfA could function together in the recognition of membrane cargoes and budding from early endosomes of vesicles allowing their recycling/targeting to specific subcompartments. The presence on AdcA of putative interaction sites with clathrin and ESCRT components raises the question of how members of the endocytic machineries interact and function with AdcA. Because AP-3 has recently been shown to co-immunoprecipitate with *Dictyostelium* ArfA [53], it will be of particular interest to investigate whether AdcA functions together with the adaptor protein AP-3 (rather than AP-2) at the surface of the macropinosomes, this all the more so as AP-3 subunits colocalize with ARF1 on mammalian endosomal structures [50] and were found in a proteomics analysis of β-arrestin interactors [54]. The identification of AdcA cargo(es) by differential proteomics analysis of endosomal compartments in the parental and *adcA* null strains is underway and should help settle this issue conclusively. Initially, β-arrestins were discovered to be GPCR regulators able to uncouple ligand-activated receptors from their associated G-proteins and to terminate downstream signaling cascades. Over the past few years, the list of targets regulated by β-arrestins has been extended to various non-GPCR transmembrane proteins such as transporters and receptor tyrosine kinases. In addition to a wide repertoire of transporters, *Dictyostelium* genome encodes no less than 55 distincts GPCRs that represent a primary list to search for AdcA targets.

## Supporting Information

Table S1 **Members of the arrestin clan in** ***D. discoideum*** **^a^.**
(DOC)

Figure S1 **Multiprotein alignment of AdcA homologs.** Homologs of *D. discoideum* AdcA were searched using blastp and aligned with the online Kalign tool (http://msa.sbc.su.se/cgi-bin/msa.cgi). The intensity of the background reflects the % of conservation of a given position within the 6 sequences (light grey>60%, dark grey>80%, black, full identity). Grey arrows indicate β strands predicted in all six sequences. The conserved FYVE domain is underlined and amino acids corresponding to the consensus signature are indicated by stars.
(TIF)

Figure S2 **Expression of AdcA during development.** Whole cell extracts from KAx-3 cells at different stages of development were analyzed by Western blot using the anti-AdcA antibody.
(TIF)

Figure S3 **AdcA_myc_ is associated to bacteria-containing phagosomes.** Cells expressing AdcA_myc_ were let to internalize TRITC-labeled *E. coli* for 1 hr. Cells were then fixed in methanol and processed for immunofluorescence with an anti-myc antibody. Optical sections were taken every 0.250 µm throughout the cell and digitally deconvolved using Axiovision software. A median z section is shown. The scale bar represents 3 µm.
(TIF)

Figure S4 **LY294002 affects AdcA endosome association.** Cells expressing AdcA_GFP_ were treated with 25 µM LY294002 or an equivalent volume of DMSO (0.05%). Live cells were observed immediately by fluorescence microscopy on a Zeiss Axiovert 200 M microscope. Pictures were acquired using Axiovision software 10 min after addition of the drug. The scale bar represents 5 µm.
(TIF)

Figure S5 **AdcA_GFP_ is associated with endocytic vesicles in the absence of the endogenous protein.** (**A**) Disruption construct. The *adcA* knock-out strain was generated by homologous recombination leading to insertion of the blasticidin resistance cassette in AdcA locus in position 497 (in bp) of its genomic DNA. The knock-out genotype was validated by Southern blot using a DIG-labeled PCR fragment (AdcA probe) to probe the EcoRV-digested genomic DNA or by Western blot on a whole cell extract of KAx-3 and *adcA* null strains using the anti-AdcA antibody. (**B**) The locations of AdcA_GFP_ and AdcAΔHФ_GFP_ were analyzed in cells lacking endogenous AdcA. Imaging was performed on a Leica TCS-SP2 confocal microscope. The scale bar represents 2 µm.
(TIF)

Movie S1 **ArfA_GFP_-binding structures are highly dynamic.** Cells expressing ArfA_GFP_ were let to adhere on coverslips in Labtek chambers. Live imaging was performed on a Zeiss Axiovert 200 M using the time-lapse module of Axiovision. Images were taken every 0.25 sec and played at a 30 frames/sec rate (7.5 fold acceleration).
(AVI)
