# Supplementary material for: FYVE-Dependent Endosomal Targeting of an Arrestin-Related Protein in Amoeba
Source: PLoS One. 2010 Dec 13;5(12):e15249. doi: 10.1371/journal.pone.0015249 (PMC3001460; doi:10.1371/journal.pone.0015249)
Supplement: Table S1 — Members of the arrestin clan in D. discoideum a. (DOC) [file pone.0015249.s001.doc]

| **Name** | **dictybase**  **Gene ID** | **Chrom.** | **Nb**  **introns** | **ESTs** | **Length**  **(aa)** | **Mw**  **(kDa)**^b^ | **pI**^b^ | **Other predicted domains** |
| --- | --- | --- | --- | --- | --- | --- | --- | --- |
| *adcA* | DDB_G0292924 | 6 | 1 | 6 | 580 | 64.5 | 8.6 | FYVE |
| *adcB* | DDB_G0274395 | 2 | 2 | 1 | 617 | 70.1 | 7.7 | C2, SAM1 |
| *adcC* | DDB_G0271022 | 1 | 1 | 10 | 654 | 74.2 | 8.1 | C2, SAM1 |
| *adcD* | DDB_G0286693 | 4 | 1 | 1 | 785 | 88.5 | 8.6 | FYVE |
| *adcE* | DDB_G0267584 | 4 | 0 | - | 564 | 63.5 | 9.4 | MIT, LIM |
| *adcF* | DDB_G0289229 | 5 | 0 | - | 1030 | 117.7 | 7.7 | - |

^a^Data were obtained in dictyBase (http://dictybase.org/) and protein sequences were analyzed directly for matches against Pfam 23.0 database at <http://pfam.sanger.ac.uk/>

^b^Average molecular weight (kDa) and pI were calculated using on-line software at <http://www.expasy.org/>
