# Supplementary figures and images for: FYVE-Dependent Endosomal Targeting of an Arrestin-Related Protein in Amoeba
Source: PLoS One. 2010 Dec 13;5(12):e15249. doi: 10.1371/journal.pone.0015249 (PMC3001460; doi:10.1371/journal.pone.0015249)

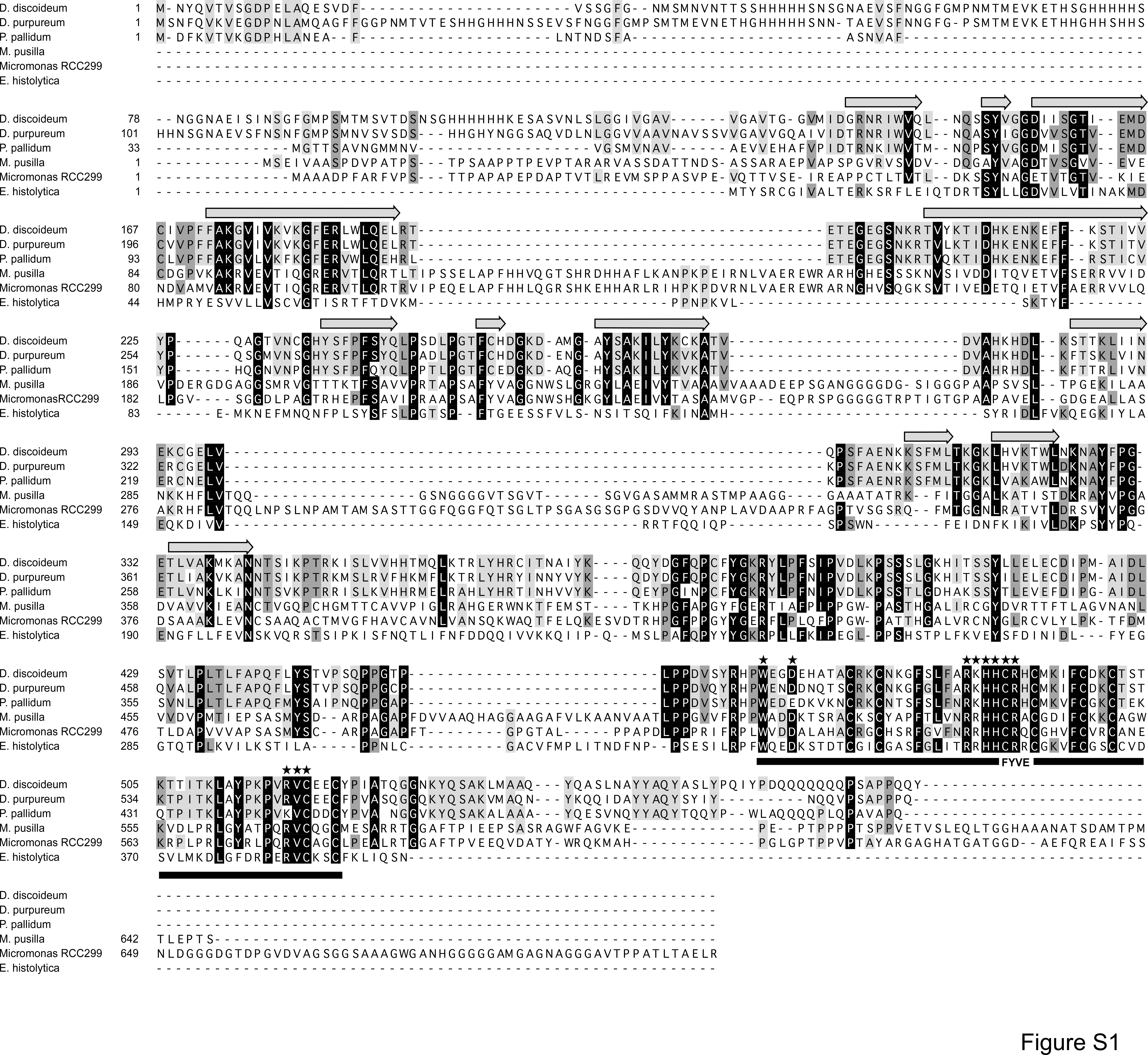

Supplement: Figure S1 — Multiprotein alignment of AdcA homologs. Homologs of D. discoideum AdcA were searched using blastp and aligned with the online Kalign tool (http://msa.sbc.su.se/cgi-bin/msa.cgi). The intensity of the background reflects the % of conservation of a given position within the 6 sequences (light grey>60%, dark grey>80%, black, full identity). Grey arrows indicate β strands predicted in all six sequences. The conserved FYVE domain is underlined and amino acids corresponding to the consensus signature are indicated by stars. (TIF) [file pone.0015249.s002.tif]

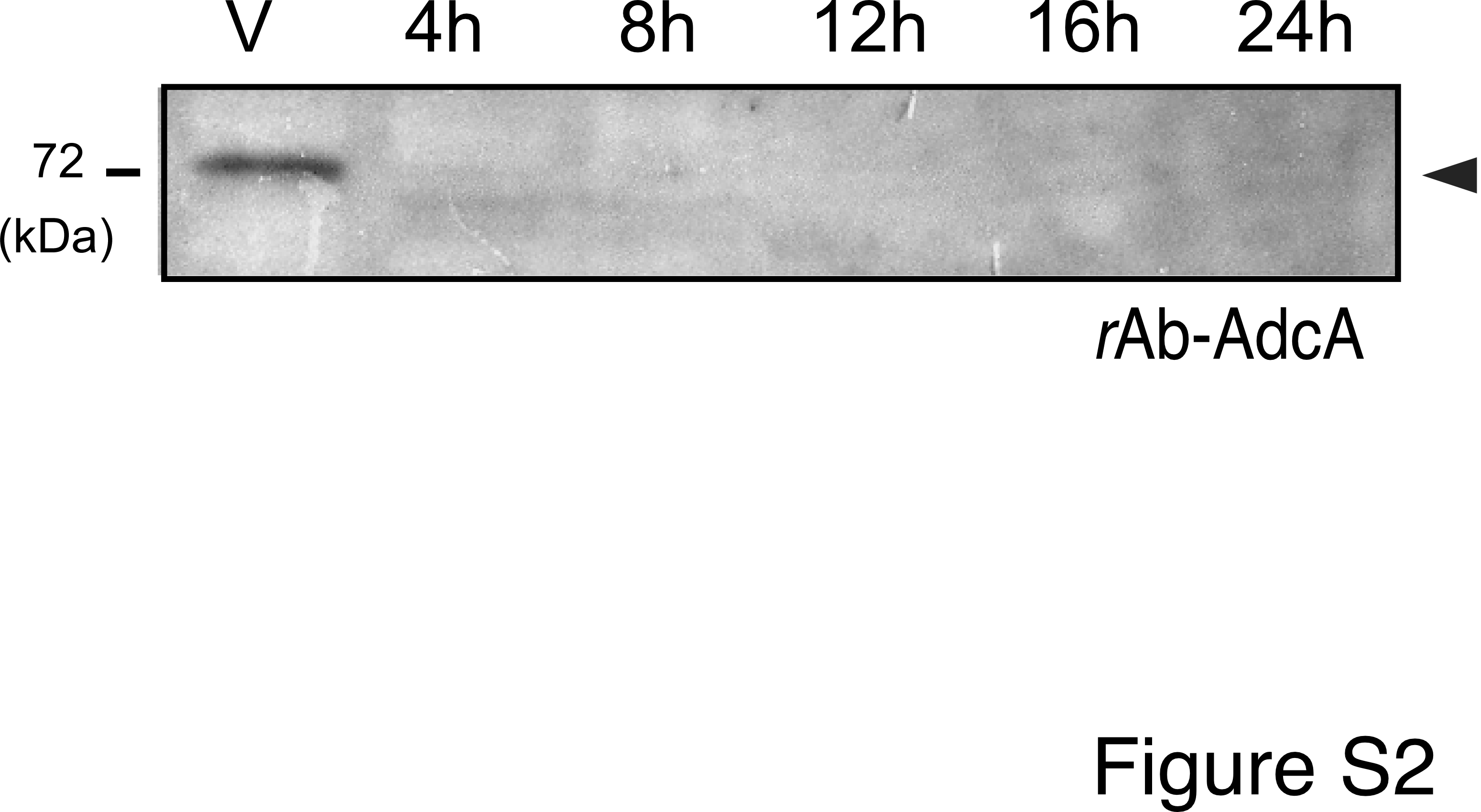

Supplement: Figure S2 — Expression of AdcA during development. Whole cell extracts from KAx-3 cells at different stages of development were analyzed by Western blot using the anti-AdcA antibody. (TIF) [file pone.0015249.s003.tif]

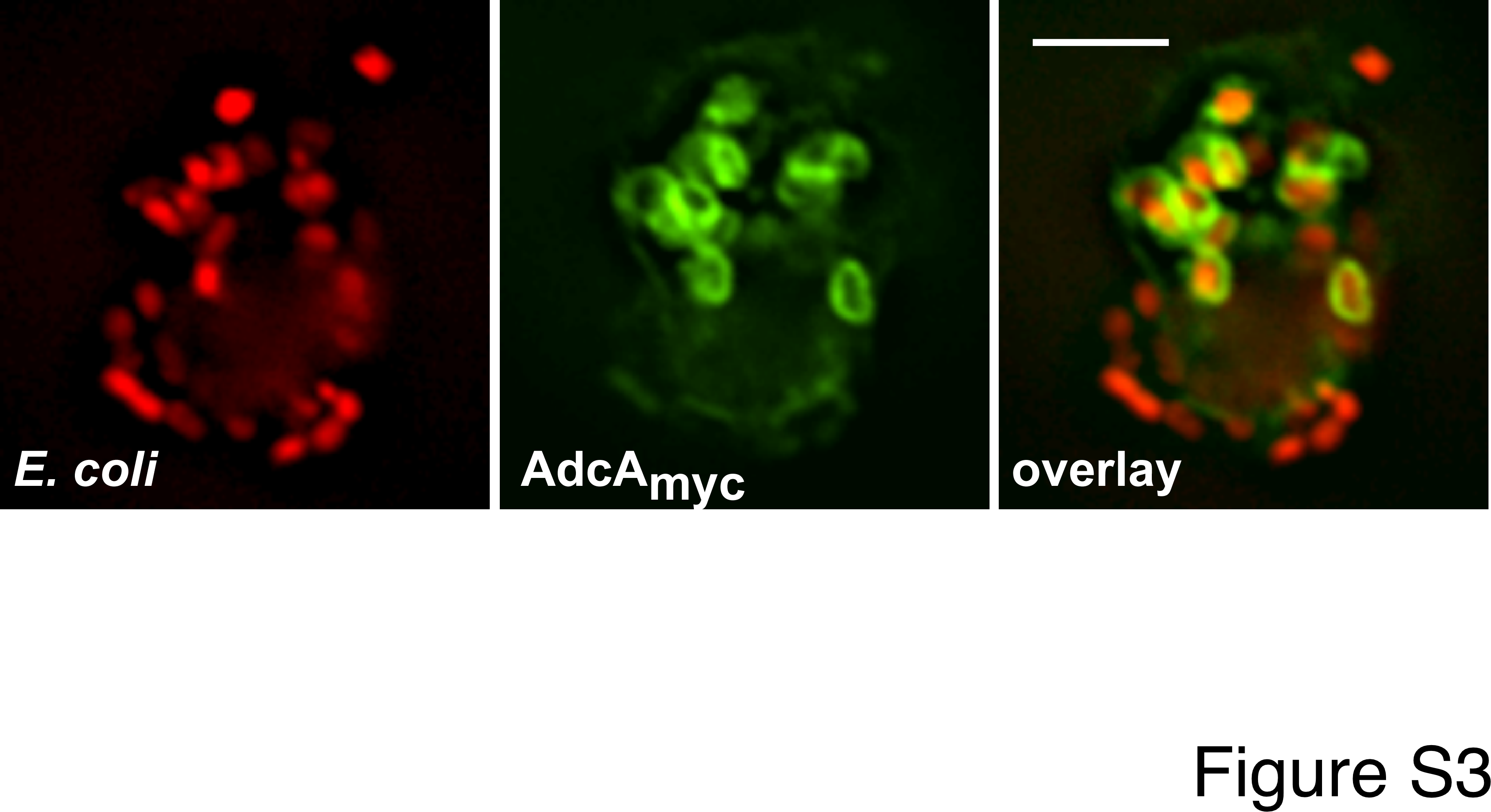

Supplement: Figure S3 — AdcAmyc is associated to bacteria-containing phagosomes. Cells expressing AdcAmyc were let to internalize TRITC-labeled E. coli for 1 hr. Cells were then fixed in methanol and processed for immunofluorescence with an anti-myc antibody. Optical sections were taken every 0.250 µm throughout the cell and digitally deconvolved using Axiovision software. A median z section is shown. The scale bar represents 3 µm. (TIF) [file pone.0015249.s004.tif]

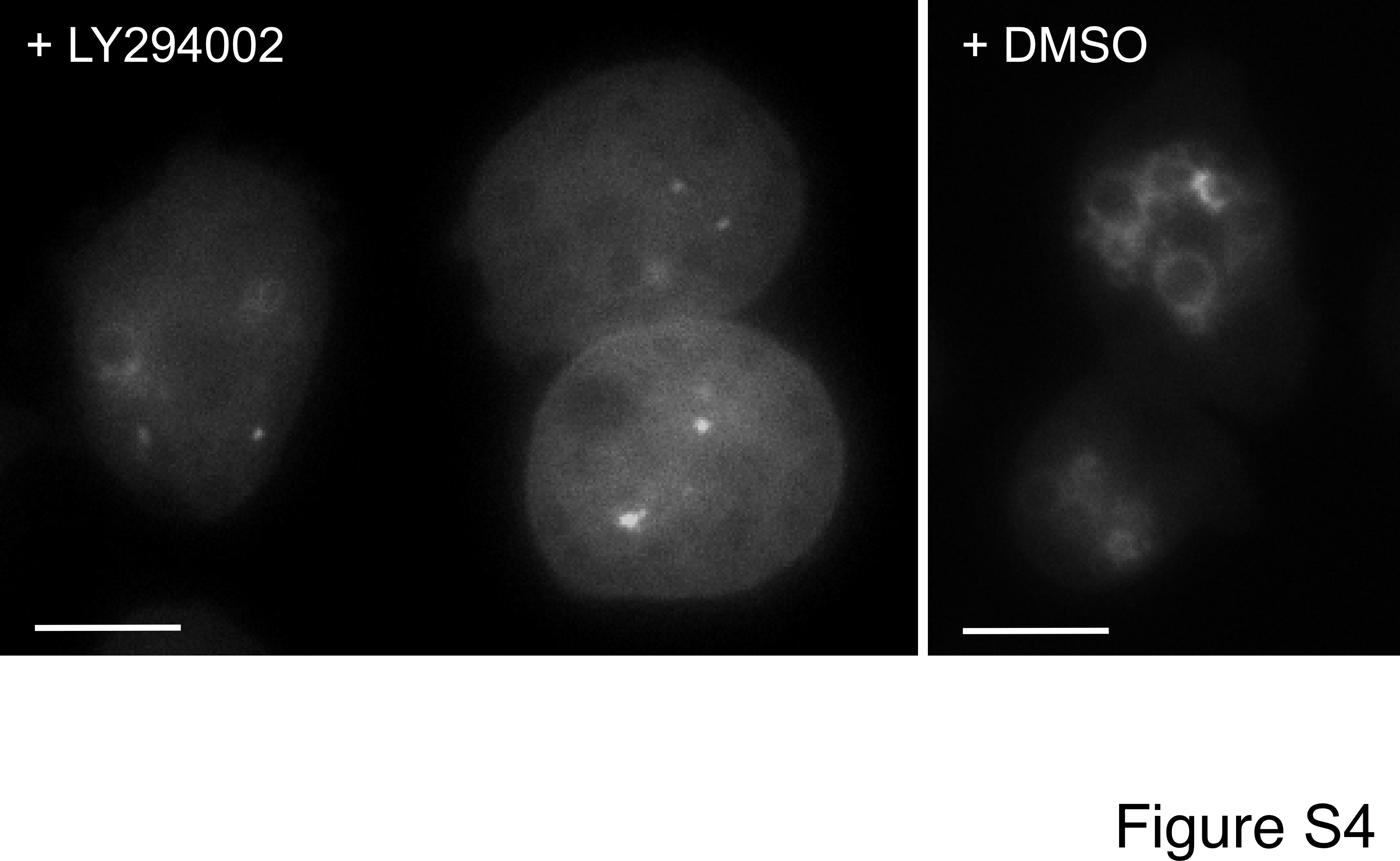

Supplement: Figure S4 — LY294002 affects AdcA endosome association. Cells expressing AdcAGFP were treated with 25 µM LY294002 or an equivalent volume of DMSO (0.05%). Live cells were observed immediately by fluorescence microscopy on a Zeiss Axiovert 200 M microscope. Pictures were acquired using Axiovision software 10 min after addition of the drug. The scale bar represents 5 µm. (TIF) [file pone.0015249.s005.tif]

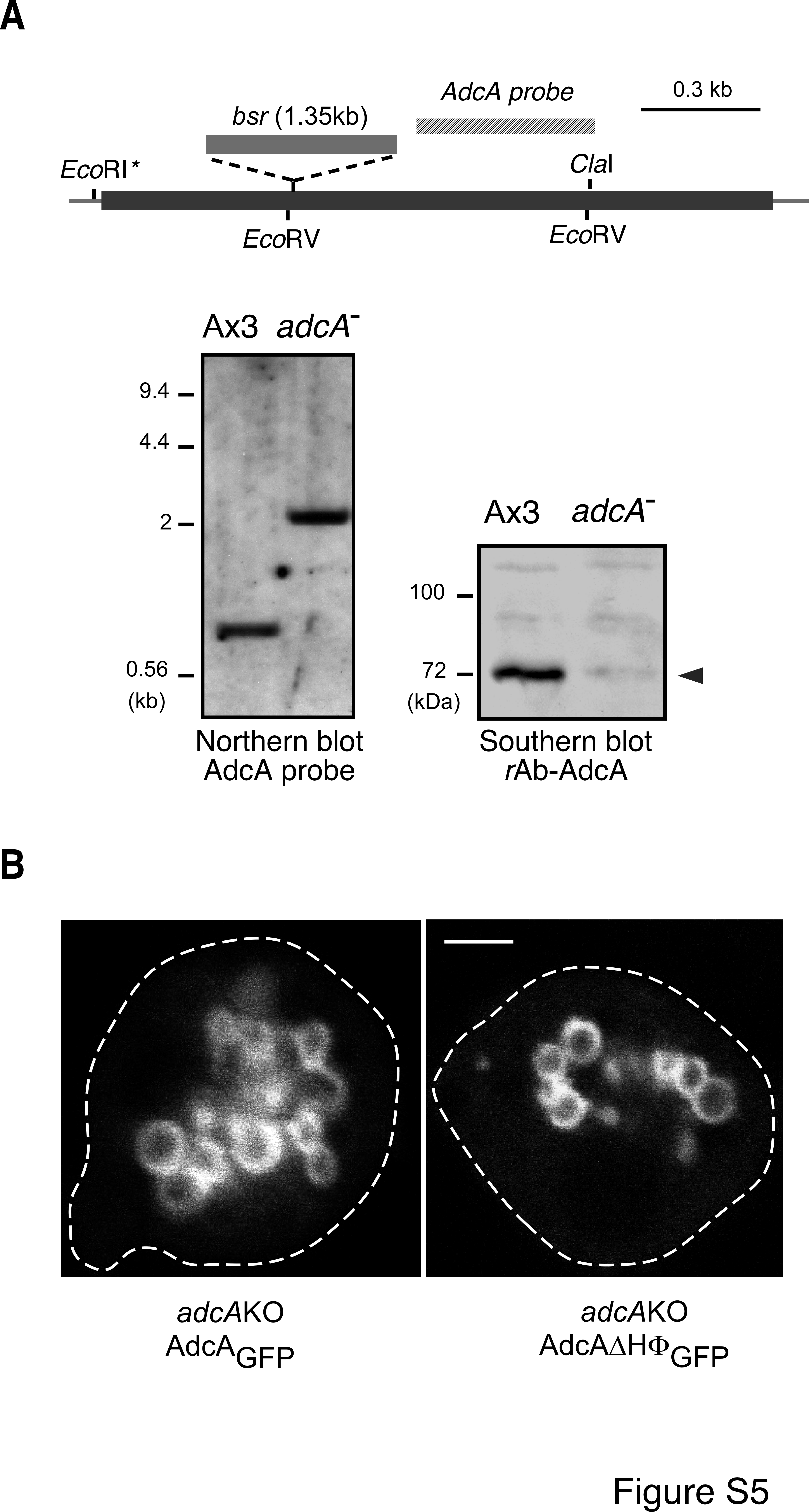

Supplement: Figure S5 — AdcAGFP is associated with endocytic vesicles in the absence of the endogenous protein. (A) Disruption construct. The adcA knock-out strain was generated by homologous recombination leading to insertion of the blasticidin resistance cassette in AdcA locus in position 497 (in bp) of its genomic DNA. The knock-out genotype was validated by Southern blot using a DIG-labeled PCR fragment (AdcA probe) to probe the EcoRV-digested genomic DNA or by Western blot on a whole cell extract of KAx-3 and adcA null strains using the anti-AdcA antibody. (B) The locations of AdcAGFP and AdcAΔHФGFP were analyzed in cells lacking endogenous AdcA. Imaging was performed on a Leica TCS-SP2 confocal microscope. The scale bar represents 2 µm. (TIF) [file pone.0015249.s006.tif]
